# Analysis of Graphene Antenna Properties for 5G Applications

**DOI:** 10.3390/s19224835

**Published:** 2019-11-06

**Authors:** Siti Nor Hafizah Sa’don, Mohd Haizal Jamaluddin, Muhammad Ramlee Kamarudin, Fauzan Ahmad, Yoshihide Yamada, Kamilia Kamardin, Izni Husna Idris

**Affiliations:** 1 Wireless Communication Centre, Universiti Teknologi Malaysia, Johor Bahru 81310, Malaysia; sitinorhafizahsaadon@gmail.com; 2 Faculty of Electrical and Electronic Engineering, Universiti Tun Hussein Onn Malaysia, Parit Raja, Batu Pahat 86400, Malaysia; mramlee@uthm.edu.my; 3 Department of Electronic Systems Engineering, Malaysia-Japan International Institute of Technology, Universiti Teknologi Malaysia, Jalan Sultan Yahya Petra, Kuala Lumpur 54100, Malaysia; fauzan.kl@utm.my (F.A.); yoshihide@utm.my (Y.Y.); kamilia@utm.my (K.K.); 4 Division of Communication Engineering, School of Electrical Engineering, Faculty of Engineering, Universiti Teknologi Malaysia, Johor Bahru 81310, Malaysia; husnaidris89@gmail.com

**Keywords:** 5G antenna, graphene antenna, CPW antenna, antenna array, defected ground structure

## Abstract

The incoming 5G technology requires antennas with a greater capacity, wider wireless spectrum utilisation, high gain, and steer-ability. This is due to the cramped spectrum utilisation in the previous generation. As a matter of fact, conventional antennas are unable to serve the new frequency due to the limitations in fabrication and installation mainly for smaller sizes. The use of graphene material promises antennas with smaller sizes and thinner dimensions, yet capable of emitting higher frequencies. Hence, graphene antennas were studied at a frequency of 15 GHz in both single and array elements. The high-frequency antenna contributed to a large bandwidth and was excited by coplanar waveguide for easy fabrication on one surface via screen printing. The defected ground structure was applied in an array element to improve the radiation and increase the gain. The results showed that the printed, single element graphene antenna produced an impedance bandwidth, gain, and efficiency of 48.64%, 2.87 dBi, and 67.44%, respectively. Meanwhile, the array element produced slightly better efficiency (72.98%), approximately the same impedance bandwidth as the single element (48.98%), but higher gain (8.41 dBi). Moreover, it provided a beam width of 21.2° with scanning beam capability from 0° up to 39.05°. Thus, it was proved that graphene materials can be applied in 5G.

## 1. Introduction

Over the next few years, researchers and scientists anticipate that the next communication technology—fifth generation cellular network technology (5G)—will have greater overall capacity and implement a new spectrum [1]. The system capacity will support users up to one thousand times more than the current level, while the spectral efficiency, energy efficiency, and data rate are capable of increasing up to ten times. Cell throughput is estimated to achieve a twenty-five times increase [2], and the cost will be more effective in terms of power usage [3]. These features are expected to allow for the usage of mobile broadband communications that would lead to a thousand-fold increase in total mobile traffic by 2020 [4]. These requirements necessitate architectural and component design changes such as device-centric architecture [5].

Much effort has been put into achieving the requirements of 5G such as broadband dual-band antennas [6], multi-input–multi-output (MIMO) antennas [7], mobile phased arrays [8], and flexible antenna arrays [9]. Specifically, in antenna design, most of the frequency spectra studied are in the millimetre wave category. This spectrum was selected in order to overcome bandwidth limitations and congestion. However, it has not been standardised; as stated in Reference [10], 5G is expected to benefit from utilising 6 GHz in its call for input (CFI) to the stakeholder. However, at the high-frequency spectrum, the demand for higher capacity can be solved by the large bandwidth it produces. Thus, the high data rate service should reach up to 1 Gb/s [11,12] which implies that the bandwidth at least 1 GHz [13]. Based on the findings in References [14,15], the application of 5G will involve approximately 70%–80% indoor communication compared to 20%–30% for outdoor. The conventional indoor antenna commonly has omnidirectional radiation and gain between −8 and 0 dBi [16]. This range of gain is too low for signal penetration, and millimetre waves experience some disturbances, for example, when the signal becomes weak during penetration of solid materials or building walls [4] and when absorbed or scattered by gases, rain, foliage [12], and flora [14]. Furthermore, in 5G antenna manufacturing, different applications require particular materials, mainly to save costs, besides the need for them to be flexible, -easy and fast fabrication, suitable for small size productions, and lightweight. Some devices may even be down-scaled [17] in size due to the higher frequency spectrum utilisation. 

Prior to this study, previous works on 5G antennas have been conducted, particularly on bandwidth, gain, and beam steering in terms of the projection of higher data rates, further distance, and reliable communications, respectively. Bandwidth improvement can be shown in many ways such as balun integration [18], coupled resonant structures [19], slot antennas [20], proximity-coupled microstrip planar arrays [21], airfield cavities [22], and corporate stacked microstrips [23]. The gain can be enhanced with double-sided bow-tie parasitics [24], lenses [25], and metal directors [26]. Another technique for gain improvement is by mutual coupling reduction. This technique can be applied between the antenna elements with the implementation of stubs [18], electronic band gap [27], defected ground structure [28], and complementary split ring resonator [29]. In order to reduce interference, the antenna array should have high directivity beam steering [13]; thus, the previous works on phased-array antennas have been reviewed in References [8,30,31,32]. 

In conjunction with the emergence of 5G, the properties of graphene have been considered in this work. Graphene is the thinnest two-dimensional layer of carbon atoms [33] bound in a honeycomb lattice [34] and exhibits the highest carrier mobility, 200,000 cm^2^ V^−1^ s^−1^ compared to other materials. It becomes an attractive material for the manufacturing of ultra-high speed electronics [17] besides offering excellent switching characteristics [35] or tunable properties [34]. The switching and tunable properties can be realised in the presence of DC voltage. In addition, graphene is categorised as semi-metal with zero bandgap which means the valence and conduction bands meet at the K points of the Brillouin zone [17] or Dirac point [36]. This property provides the opportunity for a device will never switch off, even if it uses bulk graphene. Since a graphene layer is one atom thick, it allows for unprecedented electrostatic confinement and is extremely flexible [36]. Based on the aforementioned properties, graphene is considered by researchers and scientists as a new material which can be used in electronics for radio frequency (RF) or antennas [37,38,39,40,41], sensors [42], transparent devices [43], switches [36,44,45], etc. Table 1 summarises the performance of antennas using graphene. As can be noted in Table 1, most graphene antennas working at a lower frequency are applied for radio-frequency identification (RFID), wearables, and for low-cost purposes. In contrast, when graphene antennas are designed for reflectarrays [46], reconfigurable antennas [47], tunable antennas [48], and beam scanning [49], the operating frequencies utilised are in terahertz (THz). From these studies, it can be seen that the frequencies suggested for 5G, which are between 6 GHz and 95 GHz, have not been fully investigated in in the context of graphene.

Based on the 5G requirements and limitations aforementioned, this paper proposes the development of graphene antennas in single and array which operates at a frequency of 15 GHz for 5G applications. The 15 GHz frequency was selected since the range is at high frequency. Thus, it can produce large bandwidth to support higher speed [10]. Note that the location of 15 GHz in between 6 GHz to 20 GHz was not explored as much as mm waves, since the equipment with this frequency can be obtained easily. The graphene used in this study is a new material that has potential use in future electronics due to that fact of its exclusive mechanical and electrical properties [55]. The single antenna was a coplanar waveguide (CPW)-fed rectangular slot with chamfer for easy-to-fabricate purposes. Then, the antenna array in this study consisted of four elements in order to increase gain, so that higher signal attenuation could be achieved, considering the beam steering and narrow beam [16]. The increase in gain by increasing the number of elements was studied in Reference [9]. The antenna array design introduced a defected ground structure (DGS) for reducing mutual coupling in order to reduce the sidelobe level (SLL) and improve gain. The final section of this paper presents the results of the beam steering performance.

The structure of this paper is as follows: Section 2 describes the graphene antenna design and fabrication, parametric studies, and results for the single element. The array antenna and the examination of the mutual coupling reduction, inter-element spacing, results, and beam steering are described in Section 3. The conclusion is drawn in Section 4.

## 2. Single Graphene Antenna

### 2.1. Antenna Design

**Figure 1:**
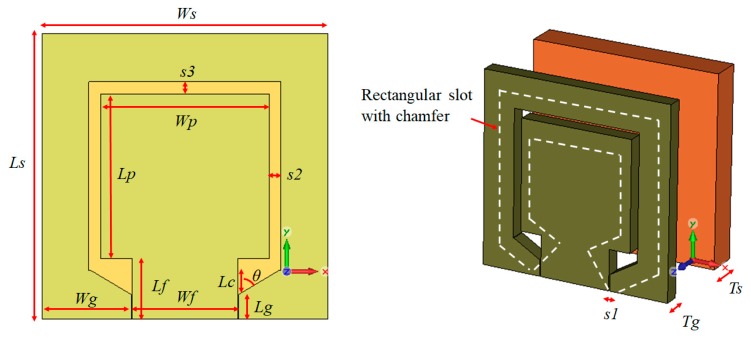
The parameter of the printed graphene antenna with CPW-fed rectangular slot with chamfer.

Figure 1 shows the graphene antenna’s parameter length, modelled on a Kapton polyimide film substrate with dielectric constant, *ε_r_* = 3.5, and tan *δ* = 0.002 at 1 kHz. The graphene used was from graphene dispersion with ethyl cellulose in terpineol, or commonly known as graphene ink, which has a sheet resistivity between 0.003 Ω.cm and 0.008 Ω.cm with a thickness near 100 nm. The antenna was printed by a screen-printing method and fed by a 50-Ω CPW. The top substrate was coated with the patch and ground plane. Then, the antenna was excited through CPW to achieve a large bandwidth [9,56], whereas a rectangular slot with chamfer was introduced to realise the impedance matching and resonance frequency required. The printed graphene antenna was manufactured to achieve the thinnest size as well as to realise the theory of downscaling.

Before optimisation, the patch width, *Wp* antenna size was estimated by Equation (1):

(1) $$W_{p}=\frac{c}{2f_{ο}\sqrt{\frac{\varepsilon_{r}+1}{2}}},$$

where *c* is the velocity of light, 3 × 10^8^ m/s, *f_ο_* is centre frequency, and *ε_r_* is the dielectric constant of a substrate. Then, the distance of the separation between the two ground planes of the waveguide (CPW), *Dcpw*, was calculated by Equation (2) [57,58]:

(2) $$D_{cpw}=2.s1+Wf,$$

where *s*1 is the slot width between the ground plane and the feedline, and *Wf* is the centre strip conductor width or feedline width.

The antenna was simulated in Computer Simulation Technology (CST) Microwave Studio. The printed graphene antenna had a width and length of 12.2 mm and 11.8 mm, respectively, and a thickness of 76 μm. The details of the antenna parameters are summarised in Table 2.

**Figure 2:**
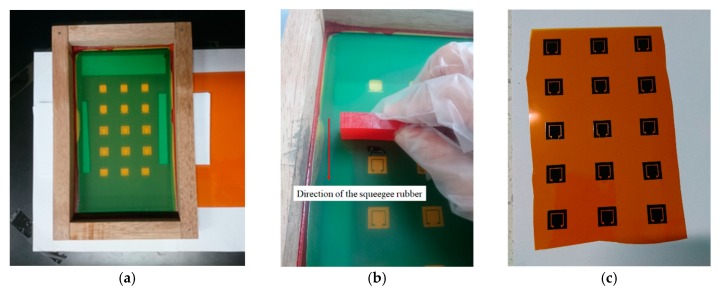
(**a**) The arrangement of the stencil and Kapton film before screen printing; (**b**) the graphene ink is printed on the Kapton film using the stencil; and (**c**) the graphene ink formed an antenna pattern after screen printing.

**Figure 3:**
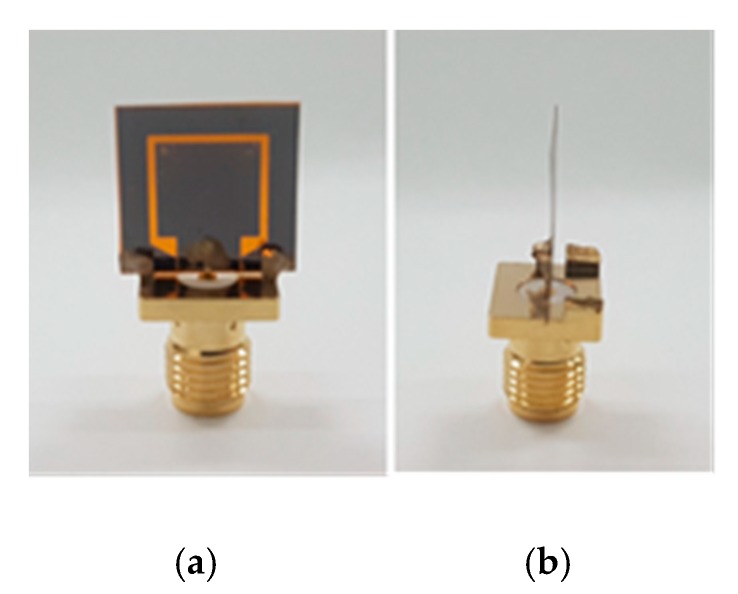
(**a**) The graphene antenna after curing and (**b**) after it is assembled with an SMA connector.

Screen printing is a technique of printing design patterns from an ink solution onto a substrate surface through a mask using a squeegee rubber movement. The method has been widely used in electronic manufacturing [51,59,60,61,62,63,64,65,66]. Screen printing offers low cost [65], high speed [66], compatibility with a variety of inks and substrates [62], an environmentally friendly process, and good accuracy [60]. In this work, screen printing was used to print the proposed graphene antenna using graphene ink on a Kapton polyimide film substrate. The screen, which is called a stencil, contains the antenna pattern with a resolution of 120 µm. Before printing, the Kapton film was placed below the stencil with a spacer at each side of the stencil. The spacer was used to avoid the film or substrate attaching to the stencil after printing. Then, a small amount of graphene ink was placed near to the antenna pattern using a disposable dropper. Printing was done by move the squeegee from the graphene ink placed towards the whole single antenna pattern. While moving, the squeegee had to be tilted and pressed at a 45° simultaneously. Figure 2a–c shows the condition during the screen-printing process, and the result after screen printing. The graphene ink was cured at 300 °C to 350 °C for 20 min to 30 min for decomposition of the binder directly in the graphene ink, increasing the graphene’s conductivity. Figure 3a,b shows the printed graphene antenna that was cut into its size and connected to a 2.92 mm diameter Sub Miniature version-A (SMA) connector.

Related to the graphene ink which needs a curing process, a Kapton film was adopted as a substrate. This was due to the film’s unique combination of properties which make it ideal for variety of applications in many different industries [67]. It also has excellent physical, electrical, and mechanical properties, because it maintains its performance over a wide range of temperatures, even as low as −269 °C and as high as 400 °C. Thus, the Kapton polyimide film is recommend and is most suitable for use with graphene ink which needs to be treated at high temperature compared to conventional substrates which are not resistance to heat.

### 2.2. Parametric Studies

Several parametric studies were executed to observe the antenna’s performance and to identify problems. There are many factors that could disturb the accuracy of the antenna’s results which happen during the fabrication process and other technical works. During fabrication, the graphene ink was handled manually; thus, the antenna produced may have an inaccurate size for small parts. During the curing process, the temperature and time set may have affected the condition of graphene and substrate. Then, after the curing process was completed, the graphene antenna was cut using penknife where small defects may have been created.

**Figure 4:**
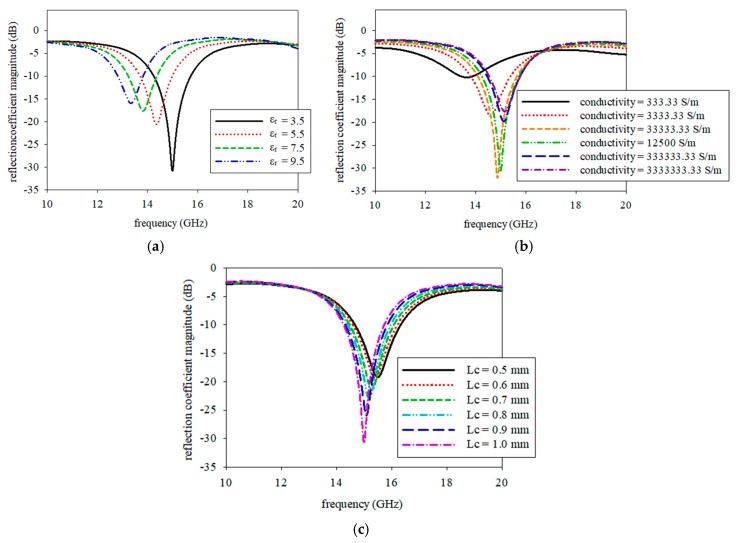
The response of the reflection coefficient and resonance frequency on the (**a**) dielectric constant of the substrate, (**b**) the conductivity of graphene, and (**c**) the cut length.

The parametric studies were the dielectric constant of the substrate, conductivity of the graphene, and cut length. These parameters were selected due to the fact of their significant influence on the frequency resonant. It was conducted by a single parameter which was varied while others were kept constant. The simulation results of the parametric studies are shown in Figure 4a–c.

Figure 4a shows an effect when dielectric constant values on Kapton substrate was changed. The resonance frequency shifted to the left about 1.65 GHz which was from 15 GHz to 13.35 GHz when the dielectric value was changed from 3.5 to 9.5. The dielectric change could be caused by the curing process on graphene that cured together with Kapton substrate within a range of temperature and time. On the other hand, the impact of conductivity on graphene was shown in Figure 4b. The increase in graphene conductivity from 3.33 × 10^2^ S/m to 3.33 × 10^6^ S/m shifted the resonance frequency to the right by approximately 1.53 GHz, from 13.65 GHz to 15.18 GHz. Conductivity variation can also be caused by the heat treatment value or length of the curing process. The parametric study on the cut length is depicted in Figure 4c. The resonance frequency moved from 15.48 GHz to 15 GHz when the cut length varied from 0.5 mm to 1 mm. The cut length, *Lc*, is a critical part of the fabrication process for the proposed screen-printed antenna.

### 2.3. Results

**Figure 5:**
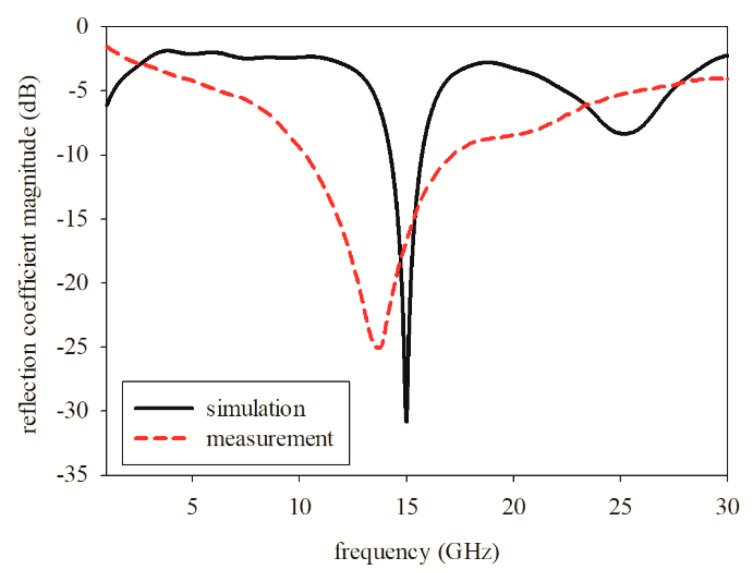
Simulation and measurement of reflection coefficient magnitude, represented by a solid curve and dashed curve, respectively.

Figure 5 shows the reflection coefficient magnitude between the simulated and measured results of the printed graphene antenna. As shown, the measured resonance frequency was 13.8 GHz, whereas the simulated was 15 GHz. The measured and simulated impedance bandwidths for reflection coefficient magnitude at −10 dB were 48.63% (10.35–17 GHz) and 9.87% (14.25–15.73 GHz), respectively. The measured resonance frequency obtained was the nearest to the simulated frequency and was the best impedance matching below −10 dB that was recorded after several fabrications and measurements. The measured resonance frequency shifted to a lower frequency due to the increase in the dielectric constant value during the curing process. It was supported by the parametric studies, where the nearest frequency that resonated at 13.8 GHz was the dielectric constant at a value of 7.5, as shown in Figure 4a, which resonated at 13.83 GHz. However, we measured the dielectric of the graphene antenna after the curing process and compared it with the simulation using the measured dielectric value which was 8.21. The simulated result showed the frequency resonated at 13.65 GHz which was also near to the measurement at 13.8 GHz. The measured results also showed a broader bandwidth compared to the simulation.

The curing process was needed to break the binder contained in the graphene ink, so that the conductivity would increase. The binder contained in graphene functioned to change the graphene flakes into ink solution and as an insulator [68]. The conductivity of the printed graphene antenna was exhibited in a number of successive iterations including the layer thickness, temperature, and time intervals involved in the curing processes [9].

**Figure 6:**
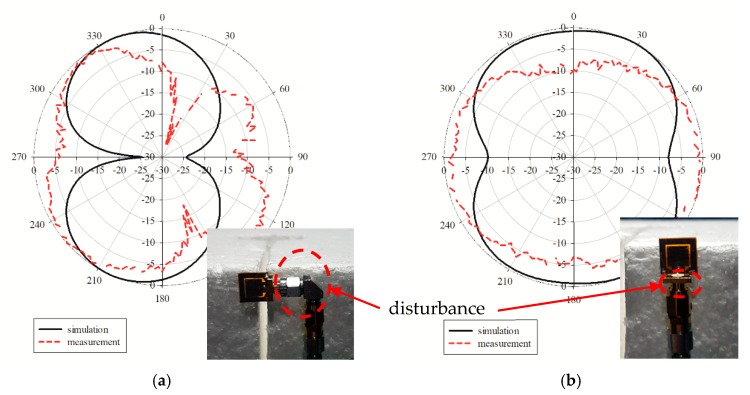
Comparison of the simulated and measured radiation patterns at (**a**) the E-plane and (**b**) H-plane.

**Figure 7:**
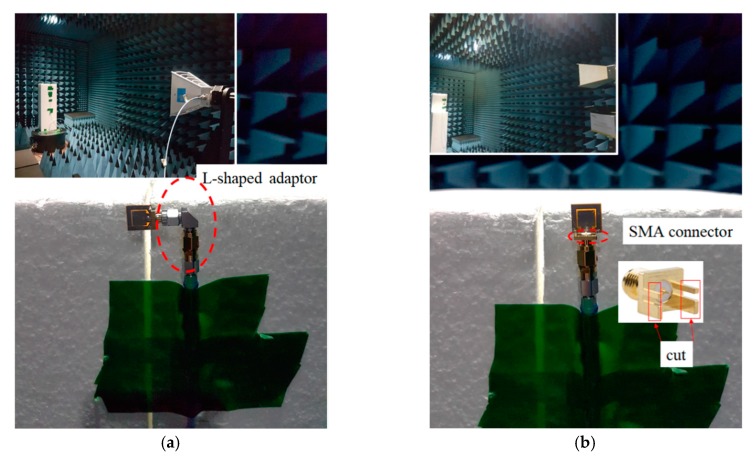
The radiation pattern measurement was read together with the (**a**) L-shaped adaptor and (**b**) the SMA connector.

Figure 6a,b shows the simulation and measurement of the radiation patterns at the E-plane and H-plane. The E-plane showed a bidirectional pattern, while the H-plane exhibited an omnidirectional pattern. Note that there were two minor lobes which appeared at the measured E-plane, while the H-plane was deteriorated. These radiation patterns were deteriorated due to the fact of several disturbances included in the complex material graphene. Since the graphene antenna was cured in a range of temperatures for a certain period, the complex conductivity of the graphene and the dielectric substrate value could not be controlled to match the values provided in the manufacturer’s data sheet. In addition, with insufficient conductivity and the changes in the dielectric value, the small size of the antenna allowed for easy disturbance by the L-shaped adaptor during the measurement of the E-plane and the SMA connector for the measurement of the H-plane as shown in Figure 7a,b. This is because the measurement reads the radiation pattern of the graphene antenna together with the adaptor and connector.

Disturbance was observed on the other type of graphene antenna with the same design and operating frequency. The two minor lobes that appeared in the radiation pattern at the E-plane for the antenna made with graphene ink also appeared in the antenna made by graphene sheet but to a smaller degree due to the higher conductivity, while, the radiation pattern at the H-plane for the graphene sheet was similar to the simulation. When graphene has a higher conductivity, it will not be disturbed by the adaptor and connector during radiation pattern measurement compared to graphene ink which does not have enough conductivity as stated in the manufacturer’s data sheet. Also, the graphene sheet does not go through a curing process which could affect the results. However, the radiation pattern of the graphene sheet was not included in this work.

With the respect to the resonance frequency in graphene ink, the simulated gain value was 2.39 dBi, while the measured gain was 2.87 dBi. The total efficiencies obtained for the simulation and measurement were 65.25% and 67.44%, respectively. The total efficiency, *e_o_*, was estimated through the calculation of the multiplication between the reflection (mismatch) efficiency, *e_r_*, and the antenna radiation efficiency, *e_cd_* [69]. The radiation efficiency is given by division of gain with directivity. The measured total efficiency was higher than the simulated one due to the higher gain in the measurement compared to simulation. This was because the measured gain was only measured at broadside radiation and at a high intensity of radiation. All the data collected are summarised in Table 3.

## 3. Antenna Array

### 3.1. Mutual Coupling Reduction

**Figure 8:**
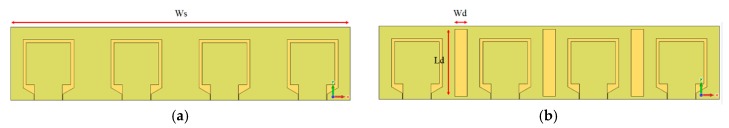
Printed graphene antenna array (**a**) before introducing the rectangular slot and (**b**) after introducing the rectangular slot.

**Figure 9:**
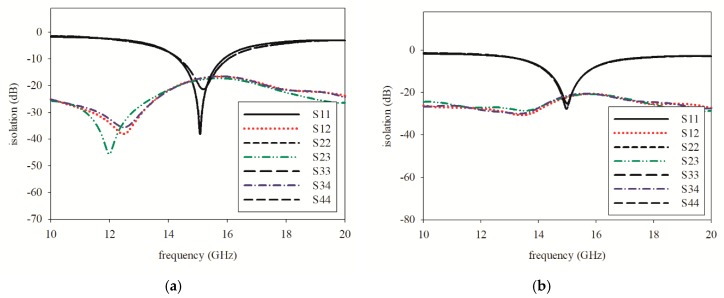
Isolation of printed graphene antenna array (**a**) before the rectangular slot and (**b**) after the rectangular slot.

**Figure 10:**
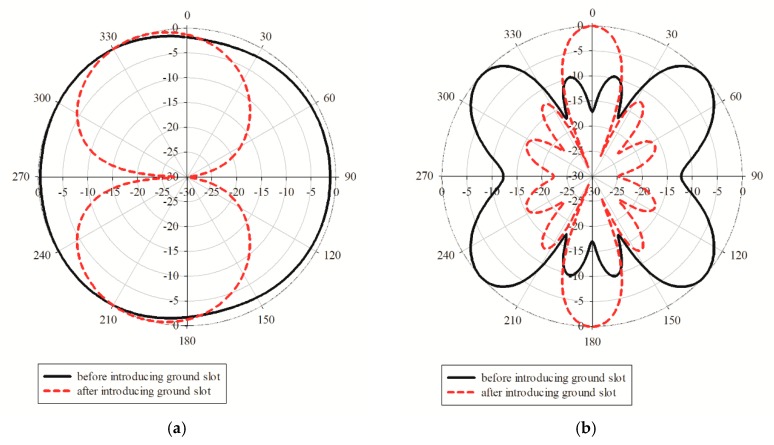
Radiation patterns of the printed graphene antenna array before introducing the rectangular slot and after introducing the rectangular slot at (**a**) E-plane and (**b**) H-plane.

Figure 8a exhibits the original printed graphene antenna array that consisted of two layers and four-element properties of the graphene antenna with a coplanar waveguide. The graphene was printed on the top layer, and the four elements were connected with a 1-to-4 power divider externally, and each ground from each adjacent element was terminated in the same layer. The antenna properties were analysed by simulation to determine the behaviour of the printed graphene antenna array. The S-parameters are presented in Figure 9a. It was observed that the printed graphene antenna array had an *S*_11_ of smaller than −10 dB from 14.32 GHz to 15.87 GHz which covered the proposed frequency bands of 5G communication, while *S*_12_, *S*_23_, and *S*_34_ were −17.47 dB at 15 GHz. However, when each element of the printed graphene antenna array was combined with the adjacent element to form a 4 element and terminate in the same ground plane, the radiation pattern at the E-plane presented an omnidirectional pattern, while the H-plane deteriorated with higher side lobe level (SLL) at −9.6 dB, as shown in Figure 10a,b, that was represented by straight line. The gain obtained at the initial design was 5.63 dBi.

According to the radiation pattern deterioration, the mutual coupling of the adjacent elements should be reduced through a technique of defected ground structure (DGS) for the improvement of the radiation pattern. Rectangular slots with a width, *Wd*, of 2 mm and length, *Ld*, of 10.8 mm in between adjacent elements were then introduced, as shown in Figure 8b, which successfully reduced the direct near-field coupling. Figure 9b shows the consequence of multiple rectangular slot architecture on mutual coupling reduction. *S*_11_ showed that the printed graphene antenna array had a frequency range of 14.30–15.71 GHz, where the impedance bandwidth was reduced to 140 MHz compared to the antenna array before introducing the rectangular slot, but it was still larger than 1 GHz which is enough for the 5G high data rate, while the mutual coupling was reduced by approximately −4.66 dB. Even though a small reduction occurred in the mutual coupling, a large effect showed up in the radiation pattern and gain, where a bidirectional pattern was exhibited in the E-plane and directive beam in H-plane, as shown in Figure 10a,b, represented by a dashed line. The H-plane had two beams at 0° and 180° and low SLL at −12.2 dB. Then, the gain increased up to 65% that was 9.28 dBi. The specifications of printed graphene antenna array before and after introducing the rectangular slot are listed in Table 4.

### 3.2. Inter-Element Spacing

The variation of the inter-element spacing or separation, *d*, and the progressive phase shift, *β*, can control the characteristics of the array factor and the total field of the array [69]. The array factor (*AF*) is given by Equation (3):

(3) $${\left(AF\right)}_{n}=cos\left[\frac{1}{2}\left(kd\;cos\;\theta +\beta \right)\right]$$

where *n* is number of elements, and *θ* is the angle observed.

**Figure 11:**
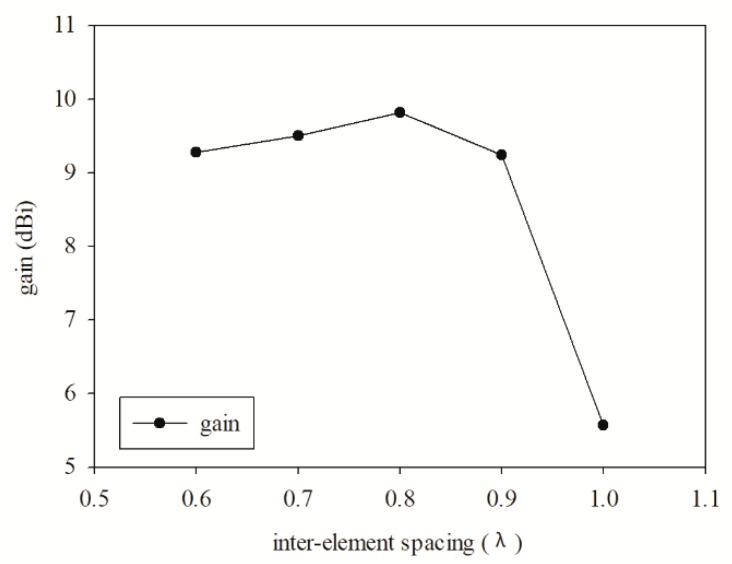
Trend of the gain value in the range of the inter-element spacing.

**Figure 12:**
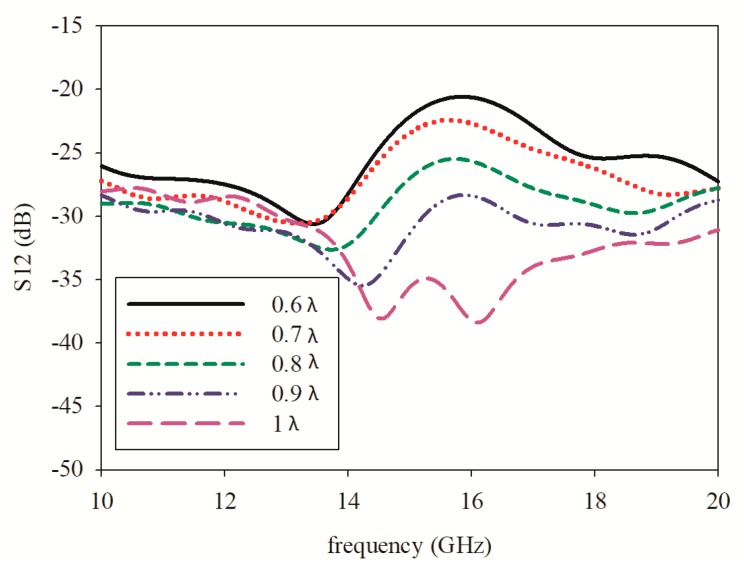
Isolation of the *S*_12_ parameter at 15 GHz with the variation of inter-element spacing.

Based on Equation (3), the inter-element spacing was observed for the effect of the gain obtained on the printed graphene antenna array after introducing the rectangular slot. The variation of inter-element spacing was between 0.6λ and λ. The gain value increased from 0.6λ to 0.8λ, then decreased until λ. The gain versus inter-element spacing is plotted in Figure 11. Meanwhile, the mutual coupling was intended to decrease with the increase in inter-element spacing as shown in Figure 12. Even though the mutual coupling was reduced at a higher inter-element spacing in between 0.9λ and λ, the gain was low due to the increase of the grating lobes. Hence, the inter-element spacing at 0.7λ was the most suitable since the gain obtained was high and the inter-element spacing was not too far compared to 0.8λ, which would increase the antenna size.

### 3.3. Results

**Figure 13:**
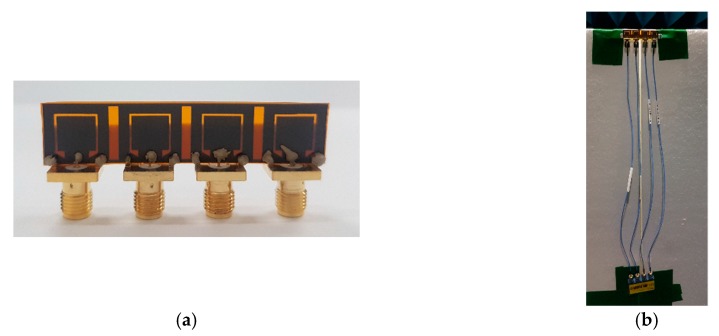
Fabricated 4 element printed graphene antenna array (**a**) with the SMA connector, (**b**) connected to *Re-Formable Semi-Rigid Cable Assemblies* and an external 1-to-4 power divider.

**Figure 14:**
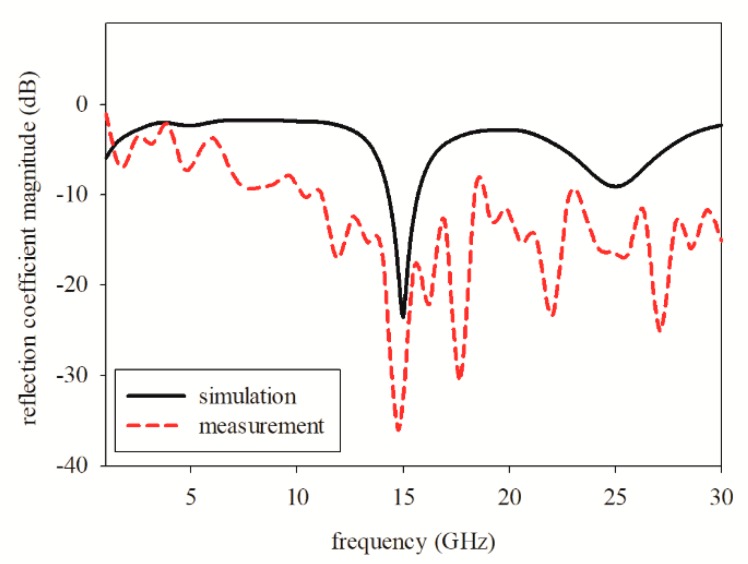
Reflection coefficient magnitude of the antenna array, showing simulated *S*_11_ in a solid curve and measured *S*_11_ in a dashed curve.

**Figure 15:**
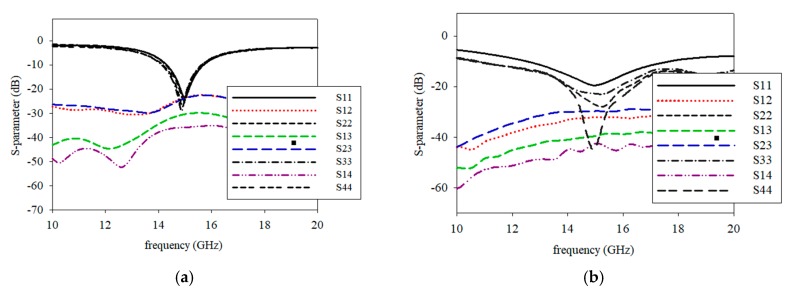
S-parameter of the printed graphene antenna array for (**a**) the simulation and (**b**) measurement.

For validation of the proposed designed, the printed graphene antenna array with a rectangular slot was fabricated as presented in Figure 13a. The antenna array was connected to an external power divider to determine the *S*_11_ measurement via a performance network analyser and then for comparison with the simulated *S*_11_ as displayed in Figure 14. It was found that both the simulated and measured *S*_11_ were less than −10 dB in the frequency bands of 14.30 GHz to 15.70 GHz and 11.10 GHz to 18.30 GHz, respectively, which are included in the proposed frequency for 5G. The resonance frequency for the measured result was approximately the same as the simulation’s result, 14.78 GHz. However, some ripples appeared in the measured *S*_11_ due to the cable loss from the *Re-Formable Semi-Rigid Cable Assemblies* that were connected between the printed graphene antenna array and the external 1-to-4 power divider as depicted in Figure 13b. The measured *S*_11_ also had broad bandwidth compared to the simulation because of the unpredicted dielectric, conductivity or composition formed by the graphene ink after the curing process. Figure 15a,b shows the simulated and measured S-parameters among the four elements, respectively. All the results presented agreed with the isolation level.

**Figure 16:**
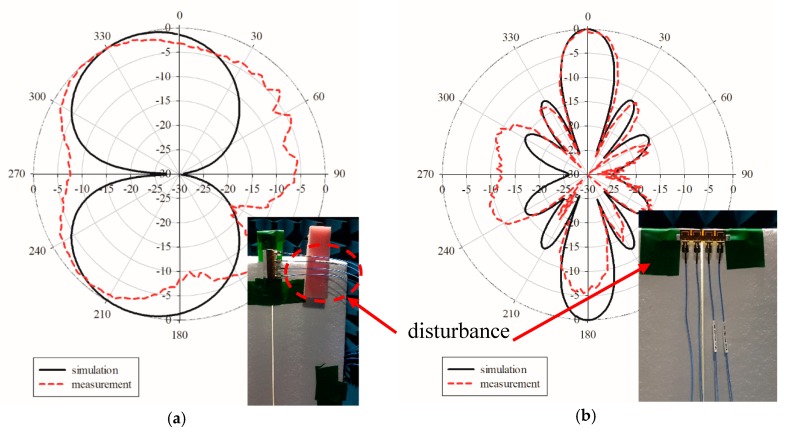
Comparison of the results of the radiation pattern of the printed graphene antenna array at (**a**) the E-plane and (**b**) H-plane.

**Figure 17:**
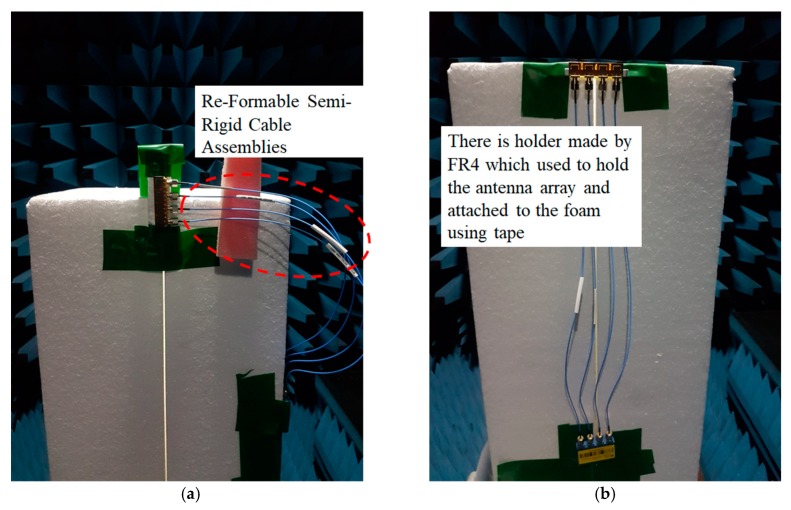
The radiation pattern measurement read together with (**a**) re-formable semi-rigid cable assemblies and (**b**) the holder and tape to attach on the foam.

The performance of the printed graphene antenna array with the rectangular slot was validated by measuring the radiation pattern. The simulated and measured normalised radiation patterns were plotted in the E-plane and H-plane at a respective frequency as shown in Figure 16a,b. It was observed that the measured radiation patterns in the E-plane of the printed graphene antenna array were nearly bidirectional as shown by the simulated radiation pattern, but the reading at 30° to 120° was not similar to that of the simulation since several *Re-Formable Semi-Rigid Cable Assemblies* were connected to the antenna array which were located at 90° during the rotation of the measurement as shown in Figure 16a. Meanwhile, the radiation pattern of the H-plane showed good agreement between the measurement and the simulation which were two directive beams at 0° and 180° except between 240° to 300° due to the tape used on the foam or disturbance by the holder when holding the antenna array at 270° as shown in Figure 16b. The holder was made by flame retardant-4 (FR-4). These disturbances are shown in Figure 17a,b. The measurements resulted in a half power beam width (HPBW) of 21.2° and an SLL of −13 dB compared to the simulation result with a HPBW bandwidth of 18.9° and −12.3 dB. From the radiation pattern presented, the measured gain achieved was 8.41 dBi and 9.50 dBi for the simulation. Table 5 presents the comparison between the simulation and measurement results of the printed graphene antenna array.

### 3.4. Scanning Performance

Theoretically, scanning phased array can be evaluated by Equation (4):

(4) $$\beta =-\;kd\;cos\;\theta_{ο}.$$

**Figure 18:**
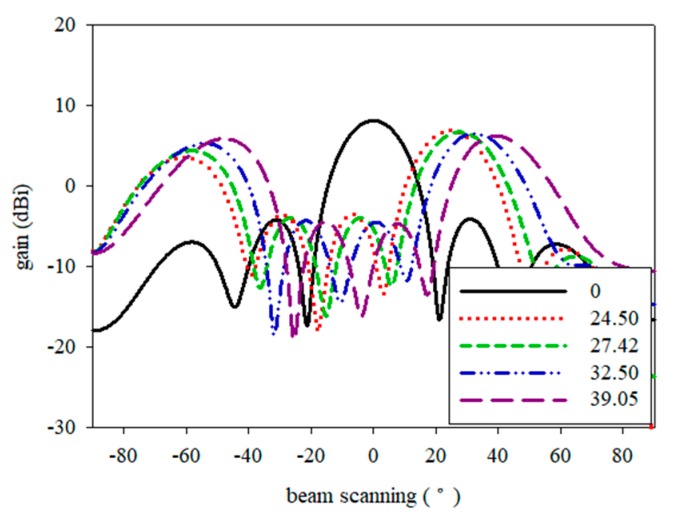
Simulation of beam scanning array.

This means that the maximum radiation can be scanned in any direction by controlling its progressive phase shifts. The scanning performance of a 4 element printed graphene antenna array with a ground slot was simulated with CST Microwave Studio. The simulated angles are listed in Table 6. The arrays were activated by four ports with the same magnitude, while the scanning was operated by the phase control at each port. The array yielded a scan angle up to 39.05° in simulation, and a gain of 6.24 dBi to 9.50 dBi. Figure 18 shows the radiation patterns of the H-plane with the scanning array at 15 GHz.

## 4. Conclusions

The performances of printed graphene antennas were studied for single and array elements at 15 GHz. The 4 element antenna array was successfully fabricated by introducing a rectangular slot for reducing mutual coupling. From the results of the impedance bandwidth, antenna gain, and antenna efficiency, the printed graphene antenna at a single element fulfils the 5G requirement, on par with the array element which has high gain, a narrow beam, and is steerable. This tiny antenna is comparable with other thin film metals and offers another selection for alternative methods to produce smaller-sized antennas. The challenge in this work was to control the dielectric substrate and conductivity of graphene during the curing process which caused a slightly different result. In the future, the graphene antenna can be improved by using a higher conductivity graphene ink or the choice of graphene ink must not involve a curing process for maintaining conductivity and dielectric stability.

## Figures and Tables

**Table 1:** The previous work on antennas made with graphene.

References	Antenna Design	Operating Frequency (GHz)	Bandwidth (GHz)	Gain (dBi)	Total Efficiency (%)	Applications
[38]	CPW-fed wideband quasi dipole antenna	1.7–5	3.3% or 98.51%	0.6–2.3	60	Low cost wireless
[50]	CPW fed slot antenna	1.73–3.77	2.04 %or 74.18%	−1–0.2	-	On-body wearable communication
[51]	Meandered line antenna	0.984–1.052	0.068% or 6.67%	−4	-	Low cost RFID and sensing
[52]	Dipole antenna	0.8–0.965	0.165% or 18.70%	−2.18	40	Low-cost and environmentally friendly RFID tags
[53]	Rectangular microstrip patch antenna	1.63	-	-	-	Wearable sensor
[54]	Microstrip antenna array	29–31	-	15.07	-	Electromagnetic shielding and radiation
Proposed antenna	CPW-fed rectangular slot with chamfer	15	(single element)	2.87	67.44	Mobile terminal for Fifth Generation (5G)
			6.63% or 48.63%			
			(array)			
			7.2% or 48.98%	8.41	72.98	

Note: “-” means not provided. RFID: radio-frequency identification; CPW: coplanar waveguide.

**Table 2:** Summary of printed graphene antenna parameter, description, and length.

Parameter	Description	Length	Parameter	Description	Length
*Ws*	Substrate width	12.2 mm	*s*1	The slot between CPW and feedline	0.08 mm
*Ls*	Substrate length	11.8 mm	*s*2	Top slot	2.5 mm
*Wp*	Patch width	7.2 mm	*s*3	Side slot	2.5 mm
*Lp*	Patch length	6.8 mm	*Tg*	Graphene thickness	100 nm
*Wf*	Feedline width	4.5 mm	*Ts*	Substrate thickness	76 µm
*Lf*	Feedline length	2.5 mm	*Lc*	Cut length	1 mm
*Wg*	Ground plane width between slot and feedline	3.77 mm	*θ*	Cutting degree	60°
*Lg*	CPW length	1 mm			

**Table 3:** Details of the printed graphene antenna’s properties for simulation and measurement.

Antenna Properties	Simulation	Measurement
Resonance frequency	15 GHz	13.8 GHz
Reflection coefficient magnitude	−30.82 dB	−25.26 dB
Frequency range at −10 dB level	14.25–15.73 GHz	10.35–17.0 GHz
Bandwidth	1.48 GHz	6.65 GHz
Percentage of impedance bandwidth	9.87%	48.63%
Gain	2.39 dBi	2.87 dBi
Total efficiency	65.25%	67.44%

**Table 4:** Specifications of the printed graphene antenna array before introducing the rectangular slot and after introducing the rectangular slot.

	Before Rectangular Slot	After Rectangular Slot
Resonance frequency	15.09 GHz	15 GHz
*S*_11_	−37.57 dB	−25.13 dB
*S*_12_	−17.47 dB	−22.13 dB
Frequency range at −10 dB level	14.32–15.87 GHz	14.30–15.71 GHz
Bandwidth	1.55 GHz	1.41 GHz
Percentage of impedance bandwidth	10.27%	9.40%
Gain	5.63 dBi	9.28 dBi
Side lobe level	−9.6 dB	−12.4 dB

**Table 5:** Detail of simulation and measurement of antenna array performance.

Antenna Properties	Simulation	Measurement
Resonance frequency	15.0 GHz	14.78 GHz
Reflection coefficient magnitude/*S*_11_	−23.50 dB	−36.02 dB
*S*_12_	−23.41 dB	−32.40 dB
Frequency range at −10 dB level	14.30–15.70 GHz	11.10–18.30 GHz
Bandwidth	1.4 GHz	7.2 GHz
Percentage of impedance bandwidth	9.33%	48.98%
Gain	9.50 dBi	8.41 dBi
Side lobe level	−12.3 dB	−13.0 dB
Half power beam width	18.9°	21.1°
Total efficiency	62.47%	72.98%

**Table 6:** Simulated beam steering performance.

Phase Shift, *β*	Beam Steering, *θ*°
0	0°
−251.72°	24.50°
−240.19°	27.42°
−221.36°	32.50°
−195.80°	39.05°
